# Mixed methods analysis of eighteen worksite policies, programs, and environments for physical activity

**DOI:** 10.1186/s12966-017-0533-8

**Published:** 2017-06-14

**Authors:** J. Aaron Hipp, Elizabeth A. Dodson, Jung Ae Lee, Christine M. Marx, Lin Yang, Rachel G. Tabak, Christine Hoehner, Oriol Marquet, Ross C. Brownson

**Affiliations:** 1 Department of Parks, Recreation, and Tourism Management, College of Natural Resources, and Fellow, Center for Human Health and the Environment, North Carolina State University, Campus Box 8004, Raleigh, NC 27695 USA; 2 Prevention Research Center in St. Louis, Brown School, Washington University in St. Louis, Campus Box 1196, One Brookings Drive, St. Louis, MO 63130 USA; 3 Agricultural Statistics Laboratory, University of Arkansas, Fayetteville, AR 72701 USA; 4 Division of Public Health Sciences, Department of Surgery, Washington University School of Medicine, 660 S. Euclid Ave., Campus Box 8100, St. Louis, MO 63110 USA; 5 Department of Epidemiology, Center for Public Health, Medical University of Vienna, Kinderspitalgasse 15, 1st Floor, 1090 Vienna, Austria; 6 BJC HealthCare, St. Louis, MO USA; 7 Department of Parks, Recreation, and Tourism Management and Center for Geospatial Analytics, College of Natural Resources, North Carolina State University, Campus Box 8000, Raleigh, NC 27695 USA; 8 Prevention Research Center in St. Louis, Brown School, Department of Surgery and Alvin J. Siteman Cancer Center, Washington University School of Medicine, Washington University in St. Louis, Campus Box 1196, One Brookings Drive, St. Louis, MO 63130 USA

**Keywords:** Obesity, Energetics, Active living, Workplace, Environment

## Abstract

**Background:**

This study examined whether specific worksite supports for physical activity (PA) were associated with total and domain-specific PA.

**Methods:**

A cross-sectional, telephone-based study was conducted in four Missouri, USA, metropolitan areas in 2012 and 2013. Outcome variables included total PA and sub-domains (leisure, work, travel) measured using the International Physical Activity Questionnaire. Logistic regression determined odds of meeting PA recommendations, given access to and use of 18 unique PA worksite supports. A subsample of 119 participants also wore hip accelerometry for seven consecutive days and maintained a wear-time diary. Access to worksite supports were associated with odds of meeting objective moderate and vigorous (MV) PA above 150 min per week.

**Results:**

Among 2013 survey participants, meeting PA recommendations while performing work-related tasks was significantly associated with several supports (e.g., walking maps, stair prompts), as was meeting recommendations during travel (e.g., flextime for PA, incentives for public transportation, walking/bicycling to work). Access to 11 worksite supports increased odds of meeting PA recommendations through leisure-time PA; five supports were associated with total PA. There were significant differences between access to and use of supports. Using objective MVPA, access to worksite challenges and bike storage were significantly associated with five and three times greater odds of meeting 150 min of MVPA per week, respectively.

**Conclusions:**

Worksite wellness plans are increasing across the US and employers are eager for evidence-based supports for increasing PA. This study provides insights into the utility of multiple worksite supports for PA to increase odds that employees meet PA recommendations.

## Background

The etiology of obesity is believed to be multi-factorial, including genetic, metabolic, behavioral, psycho-social, and environmental influences [1]. Individual behaviors that directly affect energy balance include diet and physical activity (PA), which are influenced by larger psycho-social, environmental, organizational, and policy factors. PA is associated with reduced mortality, including from cancer and cardiovascular disease, independent of its relationship to obesity [2, 3]. Unfortunately, less than half of US adults report meeting current PA guidelines [4]. If factors responsible for physical inactivity and obesity at multiple levels can be better understood, we can identify more appropriate targets for dissemination and implementation.

Many employed adults spend at least half of their waking hours at work [5]. For some, the pressures of work may impact eating and activity behaviors [6] in addition to the built environment characteristics around the worksite [7], making worksites excellent venues for health and PA promotion. Further, while the number of employees working in sedentary jobs has increased, those in physically active jobs have decreased over time [8]. This new reality highlights the importance of utilizing the worksite as an intervention site for PA promotion. A particularly promising type of worksite health promotion strategy involves environmental, programmatic, and policy changes that may assist employees in making healthful choices at work (e.g., easy access to stairways, on-site exercise facilities, time or breaks for PA during the work day) [9, 10].

Due to the rising costs of healthcare associated with obesity-related illness and disability, there is interest among employers in offering programs or benefits to assist employees in making healthful decisions [11]. This may be especially true since many chronic diseases are largely preventable, and the behaviors associated with them are often modifiable [12]. Obesity has been associated with excess costs to employers due to absenteeism, sick-leave pay, disability, and injuries [13]. Strong evidence exists that worksite health promotion and obesity programs can improve workers’ health while providing a positive return on investment [12, 14, 15].

While a variety of worksite supports for PA exist, evidence of their effectiveness is still being established [16, 17]. Available studies examine a variety of outcomes, including effectiveness of worksite supports for PA to impact overweight and obesity, musculoskeletal disorders, fatigue, blood serum lipids, blood pressure, cancer, and general health [14, 18–20]. Currently, the only worksite-specific support for encouraging PA that has sufficient evidence to be recommended by the Community Preventive Services Taskforce is encouragement of stair-use through point-of-decision prompts [21]. Thus, it is important to continue assessing what worksite supports for PA are available, to whom they are available, who is using them, and what effect they may have on increasing employee PA [18].

The overall goal of this project was to understand how environments and policies where employed adults work are associated with energy balance. In the current study, we examined whether specific types of worksite supports for PA were associated with total and domain-specific PA. Specific research questions included: 1) What access to worksite supports for PA is available at Midwestern worksites? 2) Are employees using available worksite supports? and 3) Does the presence and use of worksite supports make it more likely that employees will meet PA recommendations?

## Methods

### Participants

Participants came from the Supports at Home and Work for Maintaining Energy Balance (SHOW-ME) study, a cross-sectional, telephone-based study to understand residential environmental and worksite policy influences on employees’ obesity status and energy balance behaviors. Study design details have been previously published [22, 23].

Census tracts in four Missouri metropolitan areas (St. Louis, Kansas City, Springfield, and Columbia) were used for sampling, in an effort to achieve sample representation in racial minority and income status. Home census tracts were selected if they had a population density greater than the 10th percentile of the population density of study areas and if less than 50% of population inhabitants were 15-24 years old. The final sample was derived through a multistage, stratified sampling method that sampled participants within seven strata: metro size (large, small) and within large metro areas, walkability (low, moderate, high) and racial/ethnic minority (low, high) [24].

Between 2012 and 2013, participants were recruited, through random-digit-dialing, who met each of the following criteria: between the age of 21 and 65 years; employed outside of the home at one primary location; employed for 20 or more hours per week at one site with at least five employees; not pregnant; and no physical limitation to prevent walking or bicycling in the past week. The Institutional Review Boards of Washington University in St. Louis and the University of Missouri approved the study.

### Measurement of physical activity

The survey instrument was developed using existing self-reported and environmental assessment instruments and input from a Questionnaire Advisory Panel; significant pretesting and cognitive response testing were conducted. Survey development and testing procedures are described in detail elsewhere [23].

Primary outcome variables included total PA and PA sub-domains (leisure, work, travel) measured using the International Physical Activity Questionnaire long form (IPAQ) [25]. The IPAQ has been rigorously tested for reliability and validity [26]. For all PA variables, self-reported PA was dichotomized into meeting or not meeting national PA guidelines, according to the Centers for Disease Control and Prevention (CDC) recommendations (i.e., 150 min of moderate to vigorous PA per week) [27].

### Measurement of worksite support access and use

Worksite supports for PA included 18 unique items adapted from existing instruments. Participants were asked separately about the availability of each worksite support (e.g., ‘Does your workplace offer…’ ‘Incentives to use public transit, such as free or reduced transit pass,’ ‘Flexible time for PA during the work day for PA’). Respondents who reported presence of specific supports (for 14 out of the total 18) in their worksite were then asked about usage of the worksite support (e.g., “used in the past two months?”). A full list of supports is shown in Tables 2 and 3. Response options for worksite support questions included yes, no, and do not know. Participants who answered “do not know” were considered not to have the support available at their worksite.

### Measurement of demographic information

Participants reported data on personal characteristics, including gender, age, race/ethnicity, income, health status, self-reported health, hours worked per week, and employer size. Self-reported height and weight were used to determine obesity status.

### Measurement of objective physical activity

Participants for the accelerometry portion of the study were recruited from the pool of research participants already completing participation in the telephone survey (described above). Survey participants were eligible for the accelerometry study if they had no missing survey data on key variables of interest, resided in the St. Louis or Kansas City, MO, USA, metropolitan area, if they responded ‘Yes’ to a question asking if they would be willing to be contacted again to participate in other research studies, and had no planned overnight travel during the seven days of wear. Participants were contacted in order of survey completion date.

Accelerometer participants were instructed to wear the hip-mounted accelerometer device (ActiGraph GT3X+) during waking hours for 7 days; they were asked to re-wear the device if the accelerometer was not worn for at least 10 h per day on at least 5 days. Time periods with 90 consecutive minutes of zero counts of activity were removed from analyses [28]. An aggregate score was generated to represent the total number of minutes spent in MVPA. A cut point of 1041 counts per minute was employed to assess minutes in PA that were performed at a moderate or vigorous intensity.

Accelerometer participants also completed an activity log with daily and weekly recall items overlapping with the appropriate objective monitoring day. Participants were given one telephone call to confirm receipt of the devices and two reminder calls during the monitoring period (monitoring days 2 and 5) to ensure consent form was returned, and to prompt device wear, troubleshoot problems, and answer questions.

### Analyses

Logistic regression was used to determine the unadjusted and adjusted odds of meeting domain-specific and total PA per CDC recommendations, given access to and use of worksite supports for PA. Analyses were adjusted for race, gender, age, income, employer size, self-reported health, obesity, and hours worked per week. Due to low sample size, logistic regressions using accelerometer data were focused only on access to worksite support for PA and controlled only for employer size and income. Odds ratios and 95% confidence intervals were calculated; the threshold for statistical significance was *p* < 0.05). Analyses were conducted in R version 3.0.3. (http://www.R-project.org) and IBM SPSS v24.

## Results

Overall, 2013 people completed the survey (46% response rate of answered calls). The mean age of respondents was 48 years (standard deviation = 18 years), 67.6% were female, 62.6% were non-Hispanic White, and one-third were obese (33.7%; see Table 1 for full demographics). Nearly all (97.7%) respondents had access to at least one worksite support for PA, with 48.3% having access to at least seven of the 18 PA supports. Seventy percent of respondents reported using at least one PA worksite support. Over half of the participants reported access to water fountains *(“Do you have a clean water fountain available to you at your workplace”*), bicycle storage *(“A place to lock your bike if you choose to ride it to work”*), PA information *(“Information such as posters, brochures, emails, or lectures that encourage you to be physically active”*), and health fairs in their workplace (Table 2). Less than 15% of respondents had access to PA breaks while at work (*“Physical activity breaks conducted during meetings or at certain times of the day”*) and incentives to walk or bicycle to work (*“Incentives to walk or bike to work, such as a guaranteed ride home”*). Of those with access to the specific worksite supports, 71.2% used health fairs, 61.2% used personal services (*“Personal services, such as fitness tests or fitness or nutrition counseling”*), and 57.4% used flextime for PA (*“Flexible time for physical activity during the work day”*; Table 3). Bicycle storage was the support with the least use by those with access with only 7.1% using bicycle storage at work.

**Table 1:** Demographics of the SHOW-ME study, *n* = 2013

Characteristic	Number	Percent
Gender		
Male	652	32.4
Female	1361	67.6
Weight status		
Under or normal weight	648	33.9
Overweight	618	32.4
Obese	643	33.7
Age		
21-34	298	14.8
35-44	399	19.8
45-54	656	32.6
55-65	636	31.6
Race		
Non-hispanic white	1250	62.7
Non-hispanic black	601	30.2
Other	142	7.1
Income		
$0-29,000	391	20.7
$30-49,000	463	24.6
$50-74,000	412	21.8
$75,000 or more	620	32.9
Health Status		
Poor	33	1.7
Fair	266	13.2
Good	760	37.8
Very good	663	32.9
Excellent	290	14.4
Worksite Size		
0-49 employees	619	32.3
50-199 employees	610	31.8
200 or more employees	690	35.9
Meeting CDC recommendation of 150 min of MVPA		
No	382	19.0
Yes	1631	81.0

**Table 2:** Access to worksite supports for physical activity and adjusted odds of meeting domain-specific and total physical activity guidelines (150 min or more per week)

	N (% Yes)	Work PA aOR^a^	95% CI	Travel PA aOR^a^	95% CI	Leisure PA aOR^a^	95% CI	Total PA aOR^a^	95% CI
Personal service	1956 (48.1%)	1.09	0.87, 1.35	1.07	0.82, 1.40	1.20	0.97, 1.50	1.15	0.87,1.52
Health fair	1964 (50.6%)	1.06	0.85, 1.32	1.17	0.88, 1.54	*1.55*	*1.24, 1.94*	*1.38*	*1.04, 1.83*
Worksite challenge	1977 (46.7%)	0.98	0.79, 1.22	0.84	0.64, 1.1	*1.36*	*1.10, 1.69*	1.05	0.80, 1.38
Exercise program	1975 (36.7%)	1.02	0.82, 1.27	*1.34*	*1.02, 1.76*	*1.29*	*1.03, 1.60*	1.04	0.79, 1.37
Indoor exercise facility	2002 (35.5%)	0.96	0.77, 1.19	1.03	0.78, 1.36	*1.51*	*1.21, 1.88*	1.03	0.78, 1.36
Outdoor exercise facility	1996 (28.6%)	1.05	0.84, 1.32	*1.40*	*1.06, 1.84*	*1.26*	*1.01, 1.57*	1.19	0.90, 1.59
Shower	1973 (33.3%)	1.06	0.84, 1.32	1.13	0.86, 1.49	*1.32*	*1.06, 1.65*	1.30	0.98, 1.73
Bike storage	1909 (58.1%)	1.07	0.86, 1.32	1.24	0.95, 1.61	*1.34*	*1.09, 1.65*	*1.51*	*1.15, 1.97*
Flextime for PA	1981 (35.6%)	0.87	0.71, 1.07	*1.43*	*1.11, 1.84*	*1.44*	*1.18, 1.77*	1.22	0.93, 1.59
PA Break	1979 (14.9%)	*1.82*	*1.36, 2.43*	*1.95*	*1.43, 2.65*	1.29	0.98, 1.70	*1.65*	*1.11, 2.45*
Membership	1883 (31.2%)	.099	0.79, 1.24	1.07	0.81, 1.43	*1.39*	*1.11, 1.75*	1.21	0.91, 1.62
Incentives to bike/walk	1949 (8.6%)	1.16	0.80, 1.69	*2.10*	*1.43, 3.07*	*1.80*	*1.25, 2.60*	1.63	0.96, 2.75
Incentives for public transit	1923 (18.5%)	1.00	0.76, 1.32	*1.60*	*1.16, 2.19*	1.20	0.91, 1.57	1.39	0.97, 1.98
Walking maps	1962 (18.9%)	*1.58*	*1.21, 2.06*	1.30	0.96, 1.77	1.28	0.99, 1.67	*1.57*	*1.1, 2.25*
Stair prompts	1979 (21.8%)	*1.72*	*1.33, 2.22*	*1.45*	*1.08, 1.93*	1.25	0.97, 1.59	1.35	0.98, 1.87
PA Posters	1982 (50.4%)	1.15	0.93, 1.43	0.93	0.71, 1.21	*1.26*	*1.02, 1.56*	*1.38*	*1.06, 1.82*
Water fountain	1993 (87.2%)	1.02	0.75, 1.38	1.22	0.82, 1.81	1.32	0.97, 1.79	1.11	0.72, 1.19
Water cooler	2003 (49.9%)	0.99	0.81, 1.2	1.09	0.85, 1.39	1.02	0.83, 1.24	0.92	0.72, 1.19

^a^Adjusted for race, gender, age, income, employer size, self-reported health, obesity, and hours worked per week. All italicized adjusted odds ratios are significant at *p*<0.05. Sample sizes (N) are the number of participants responding yes, no, or do not know to *access* of the worksite support. The % Yes is the numerator yes over the denominator yes + no +do not know

**Table 3:** Use of worksite supports for physical activity and adjusted odds of meeting domain-specific and total physical activity guidelines (150 min or more per week)

	N (% Yes)	Work PA aOR^a^	95% CI	Travel PA aOR^a^	95% CI	Leisure PA aOR^a^	95% CI	Total PA aOR^a^	95% CI
Use personal service	941 (61.2%)	0.75	0.56, 1.02	1.16	0.79, 1.70	*1.41*	*1.04, 1.90*	1.02	0.70, 1.50
Use health fair	990 (71.2%)	0.96	0.70, 1.32	1.24	0.83, 1.86	1.26	0.92, 1.73	1.05	0.70, 1.59
Use worksite challenge	923 (48.3%)	0.76	0.57, 1.02	1.37	0.93, 2.01	*1.53*	*1.14, 2.06*	1.14	0.79, 1.64
Use exercise program	724 (33.6%)	*1.88*	*1.31, 2.69*	1.47	0.97, 2.23	*1.65*	*1.15, 2.37*	*2.00*	*1.24, 3.23*
Use indoor exercise facility	711 (38.1%)	*1.51*	*1.07, 2.13*	1.34	0.86, 2.08	*2.25*	*1.55, 3.25*	*2.67*	*1.64, 4.36*
Use outdoor exercise facility	570 (41.8%)	1.26	0.86, 1.86	0.83	0.52, 1.31	1.26	0.86, 1.85	*1.99*	*1.19, 3.34*
Use shower	656 (21.3%)	1.44	0.93, 2.22	1.47	0.92, 2.42	*1.83*	*1.16, 2.89*	*3.73*	*1.65, 8.43*
Use bike storage	1110 (7.1%)	1.07	0.62, 1.84	*4.38*	*2.55, 7.52*	*2.47*	*1.36, 4.49*	2.78	0.85, 9.14
Use flextime for PA	706 (57.4%)	*1.44*	*1.02, 2.03*	1.43	0.95, 2.15	*2.00*	*1.43, 2.79*	*2.28*	*1.46, 3.55*
Use PA Break	295 (71.2%)	*1.87*	*1.00, 3.51*	1.18	0.63, 2.21	1.26	0.71, 2.25	*3.03*	*1.36, 6.75*
Use membership	588 (24.5%)	1.02	0.66, 1.56	0.77	0.44, 1.35	*2.61*	*1.66, 4.11*	*2.03*	*1.08, 3.82*
Use incentives to bike/walk	167 (26.9%)	0.83	0.32, 2.12	2.03	0.90, 4.58	1.96	0.81, 4.71	1.29	0.34, 4.81
Use incentives for public transit	355 (32.7%)	1.12	0.64, 1.96	1.30	0.71, 2.39	1.09	0.63, 1.88	1.28	0.59, 2.74
Use Walking maps	370 (31.6%)	1.18	0.70, 2.00	*1.94*	*1.10, 3.42*	*2.02*	*1.18, 3.44*	2.04	0.94, 4.42

^a^Adjusted for race, gender, age, income, employer size, self-reported health, obesity, and hours worked per week. All italicized adjusted odds ratios are significant at *p*<0.05. Sample sizes (N) are the number of participants responding yes to *access* to the worksite support. The % *use* is the numerator yes *use* over the denominator yes *access*

Meeting CDC recommendations for PA while performing work-related tasks (work domain) was significantly associated with access to PA breaks, walking maps at work *(“Maps or signs of walking routes within the workplace or offsite in the neighborhood surrounding the workplace”*), and stair prompts (*“Signs encouraging the use of stairs”*; Table 2). Greater than 150 min of work-related PA was also associated with use of work-supported exercise programs (*“Regular exercise programs, such as aerobic classes, team sports, walking groups, etc.”*), indoor exercise facilities at work (*“Indoor exercise facilities, such as a workout room/gym, exercise equipment”*), using flextime for PA, and using PA breaks while at work (Table 3).

Meeting CDC recommendations for PA during travel was associated with access to outdoor exercise facilities at work (*“Outdoor exercise facilities, such as a walking path or basketball hoop”*), flextime for PA, PA breaks while at work, stair prompts, incentives for using public transportation (*“Incentives to use public transit, such as free or reduced transit pass”*), and incentives to walk and/or bike to work (Table 2). Having access to incentives to walk or bicycle to work was the least common worksite support for PA, but was associated with over a two-fold increase in the odds of meeting 150 min of weekly PA during commute and travel alone. Use of bicycle storage at work, the least used support, had over a four-fold increased odds in meeting CDC recommendations (Table 3). Using walking maps around the worksite was also significantly associated with meeting requirements in the travel domain.

Access to 11 worksite supports were associated with increased odds for meeting CDC recommendations for PA in the leisure domain (Table 2). Incentives for walking and/or bicycling to work, access to a health fair, and access to an indoor exercise facility were associated with the greatest odds of meeting PA recommendations. Use of nine worksite supports were associated with increased odds for meeting leisure-time PA recommendations. Use of a gym membership (*“Free or reduced membership to an offsite exercise facility, such as the YMCA or Bally’s”*), bicycle storage, indoor exercise facility, worksite walking maps, and flextime for PA each increased the odds of meeting PA recommendations by at least two-fold (Table 3). Access and use of worksite challenges (*“Worksite challenge events to encourage exercise or weight loss. For example, Biggest Loser- type competitions or step-count competitions”*) was only significantly associated with leisure-time PA, 36% and 53% increased odds, respectively.

For total PA (non-domain specific, the sum of all PA over seven consecutive days), access to five of 18 worksite supports were associated with statistically significantly higher odds of meeting CDC recommendations for PA. Access to PA breaks while at work, worksite walking maps, and bicycle storage each increased the odds of meeting PA recommendations by at least 50%. Seven of 14 worksite supports were associated with increased odds of meeting CDC guidelines for PA when used at the worksite. The use of a shower at work (*“Shower facilities that you can use”*) and use of PA breaks while at work increased the odds of meeting PA recommendations by over 300%.

Only three worksite supports were significantly associated with 150 or greater minutes of total PA as measured by hip-mounted accelerometry (Table 4). These were access to worksite challenge events, availability of bike storage, and access to a water cooler (*“water cooler or bottled water available free of charge at all times”*). Access to a worksite challenge was associated with an over six-fold increase in the odds of meeting PA recommendations, while having bike storage access increased odds by four times. These significant associations were found both when controlling for employer size and participant income. Three worksite supports could not be tested due to fewer than 20 participants having the support and accelerometer data (incentives to walk or bicycle to work, PA breaks, and walking maps).

**Table 4:** Access to worksite supports for physical activity and adjusted odds of meeting total physical activity guidelines (150 min or more per week) measured with accelerometer

	N (% Yes)	Model 1 aOR^a^	95% CI	Model 2 aOR^b^	95% CI
Personal service	45 (40.1%)	0.32	0.72, 1.45	0.25	0.05, 1.11
Health fair	55 (49.1%)	2.48	0.65, 9.35	2.15	0.58, 7.84
Worksite challenge	53 (46.7%)	*5.35*	*1.15, 24.8*	*6.66*	*1.39, 31.8*
Exercise program	43 (38.3%)	1.19	0.24, 5.77	0.82	0.17, 3.87
Indoor exercise facility	34 (30.3%)	0.82	0.22, 5.79	1.40	0.43, 4.54
Outdoor exercise facility	28 (25.0%)	1.61	0.46, 5.62	1.79	0.50, 6.27
Shower	37 (33.3%)	2.16	0.64, 7.26	1.76	0.54, 5.70
Bike storage	58 (51.7%)	*3.79*	*1.30, 11.0*	*4.02*	*1.36, 11.8*
Flexible time for PA	37 (33.0%)	1.15	0.41, 3.27	1.07	0.37, 3.03
Membership	23 (20.5%)	0.41	0.9, 1.84	0.71	0.19, 2.60
Incentives for public transit	21 (18.7%)	0.27	0.06, 1.30	0.21	0.04, 1.01
Stair prompts	21 (18.8%)	4.02	0.74, 21.83	2.45	0.52, 11.4
PA Posters	51 (45.5%)	0.30	0.1, 1.54	0.24	0.04, 1.19
Water fountain	91 (81.2%)	0.89	0.26, 3.12	1.25	0.36, 4.27
Water cooler	40 (35.7%)	*3.30*	*1.08, 10.1*	*3.19*	*1.09, 9.19*

Sample sizes (N) are the number of participants responding yes, no, or do not know to *access* of the worksite support. All italicized adjusted odds ratios are significant at *p*<0.05. The % Yes is the numerator yes over the denominator yes + no +do not know

^a^Adjusted for employer size ^b^Adjusted for income

## Discussion

In this study, access to and use of specific worksite policies, programs, and environments for PA increased the likelihood that employees would meet the CDC recommendation of 150 min of PA per week. In general, the *use* of supports had greater associations with PA than mere *access* to supports, suggesting future research and intervention efforts should be primed, where feasible, to move employees from awareness of supports for PA to regular use of the supports.

The association of worksite supports with PA differed by PA domain. Actual worksite or occupational PA was significantly associated with using PA breaks while at work, flextime for PA, indoor exercise facilities at the worksite, and exercise programs. The availability of several worksite supports that may be implemented for no- or low-cost (e.g., PA breaks, walking maps, and stair prompts) were found to be associated with an increased odds of work or occupational PA. Stair prompts, which have been shown to be efficacious in increasing activity, may act through nudging employees to use the stairs [21]. At a policy level, PA breaks can reflect the culture of an organization, by allowing employees to be active during the work day. Though the IPAQ-Long Occupational PA measures (*“Physical activity you did in the last 7 days as part of your paid work. This does not include traveling to and from work”*) have been shown to be moderately reliable in comparison to accelerometer-measured occupational PA, there may be on-going content validity challenges [29]. Using PA breaks while at work and using indoor work exercise facilities certainly could increase PA while at work, but these behaviors are not occupational-related PA. The addition of accelerometer-based measures of PA should assist in overcoming this limitation. However, in the present study only ten of the 112 accelerometer participants achieved 150 min of PA while at work, as defined by their time diary.

The presence and use of several worksite supports were associated with increased odds of attaining PA from travel. The positive associations, which might have been anticipated, were between the use of bike storage and walking maps and the presence of incentives for biking, walking, and using public transit. It is possible that employers offering these incentives are encouraging their employees to use alternative means of transport, which allows employees to achieve significant PA during this transport. Others have documented this relationship between similar worksite supports and active commuting. For example, Kaczynski and colleagues report that employees were more likely to walk or bike to work when they believed that coworkers did so, and when bike storage facilities were present at the worksite [30].

Employees with access to PA breaks and flextime for activity were more likely to meet PA recommendation through travel, as these breaks may allow employees to travel actively to destinations around the worksite (either for activity or not). Several supports, which were associated with travel PA, might not have been anticipated, these include stair prompts and availability of indoor and outdoor exercise facilities. It is possible that this is related to worksite culture, and the presence of these supports creates an active culture, where employees are encouraged to use active travel. Our team has found that seeing co-workers being active and agreeing that their company values health are both associated with increased PA [31].

The presence and use of many supports were associated with increased odds of meeting PA requirements from leisure-time PA, indicating a relationship between the worksite environment and activity outside of work and travel. Others have found associations between leisure-time PA and worksite PA facilities and subsidized health club memberships [32]. While the current cross-sectional study cannot determine the direction of this association, those planning worksite efforts might be encouraged that worksite programs, facilities, and policies may impact activity away from work. Of perhaps of equal interest are the few supports which were not found to be related to leisure-time PA. These included personal services for fitness, PA breaks, walking maps, stair prompts, and incentives for public transit. However most of these supports (i.e., PA breaks, walking maps, stair prompts, and incentives for public transit) were found to be related to travel PA, suggesting some supports may be more strongly associated with PA in one domain over another.

For total PA, the presence of several supports were associated with achieving recommended PA levels, including health fairs, bike storage, PA breaks, walking maps, and PA posters. Worksite challenges and bike storage availability were also found to be associated with total PA when analyzed with accelerometer data. Many of these supports could be implemented with little cost, possibly making them more appealing to employers (e.g., PA breaks and walking maps). However, some of these supports have not been shown to be affective in other literature and/or settings, so these findings are surprising [17, 33, 34]. It is possible that the presence of these supports, such as health fairs, may indicate an effort on the part of an employer to promote health generally, efforts which may be perceived by employees. Several of the supports for which use was associated with activity (i.e., exercise programs, indoor and outdoor exercise facilities, showers at work, flextime for PA, and memberships) could be directly related to intentional, planned exercise, rather than incidental PA attained throughout the day.

While no worksite supports were significantly associated with increasing PA across all three PA domains and total PA, a few were close. Specifically, access to PA breaks increased the odds of meeting PA recommendations in all domains except leisure-time PA (OR = 1.29, 95% CI = 0.98, 1.70). Only 14.9% of participants reported having access to this support at their worksite suggesting, especially given its low cost to employers, it is being underutilized. Similarly, the use of exercise programs at the worksite and the use of flextime for PA were each associated with increased odds of meeting PA recommendations in all domains except travel PA, where they achieved close to statistical significance (OR = 1.47, 95% CI = 0.97, 2.23 and OR = 1.43, 95% CI = 0.95, 2.15, respectively for access and use). Given the potential of these supports to increase the odds of meeting PA recommendations in a variety of ways, encouragement of their availability and use should be particularly emphasized.

Several worksite supports were only associated with increased odds of meeting PA recommendations in a single domain. For example, access to worksite challenge events, indoor exercise facilities, showers, and subsidized health club memberships were only significantly associated with meeting PA recommendations through leisure-time PA. Though it may be expected that such supports would contribute to leisure-time PA because each incentivizes or facilitates PA during non-work time, understanding why these supports were not associated with total PA could help shape future research efforts. Understanding the lack of associations between the use of other worksite supports and the various PA domains should be the subject of additional study, especially given their current popularity and frequent combination with wearable technologies such as Fitbits™.

Finally, worksite challenges were found to be associated with self-reported leisure-time PA and accelerometer-based objective total PA. Access to worksite challenges was found to be associated with an over 6.5-fold increase in the odds of meeting objective total PA greater than 150 min per week. This provides further, and updated support to the Community Guide which only lists the vague “worksite programs” as a tool for decreasing obesity (https://www.thecommunityguide.org/findings/obesity-worksite-programs). In this recommendation, the Community Guide notes supports such as worksite facilities for PA, but lacks further specificity.

Discussion of the importance and utility of implementing these and other worksite supports for PA should also include consideration of the potential return on investment (ROI) for employers. Recent reports suggest the extent to which worksite health programs provide a positive ROI depends on several factors, including methodological quality of studies reporting ROI of health promotion programs and the methods used to calculate ROI [35, 36]. A systematic review reported an overall weighted-ROI of 1.38, indicating a 138% return on investment [36]. Another meta-analysis calculated an ROI of 3.27 for medical costs and 2.73 for absenteeism [37]. Important methodological and measurement issues remain; for example, the quality and comprehensiveness of the worksite wellness programs included in these other studies are not available or were not assessed. Nevertheless, these reports should reassure employers that efforts to implement worksite supports to encourage employee PA will be rewarded in a positive ROI.

The present study is subject to some limitations. Though the measures used in this study are reliable, data were self-reported and thus, are subject to bias. To help validate the results obtained through the self-reported data, accelerometer data was added. In that regard, the small sample size available and the fact that only total PA could be tested pose limitations. The sampling strategy used and limited geographic area may limit generalizability of findings.

Due to the cross-sectional nature of the study, we can only observe associations and are not able to attribute causality. For example, we cannot assess if participants were already meeting PA recommendations before a worksite support was implemented. It is also possible some participants may have selected their place of employment based on the PA benefits and known worksite supports for PA. Active employees may also just be more likely to be aware of available PA supports. Such employees may be highly motivated to meet PA recommendations and their doing so may not be due solely to the presence of worksite supports for PA. A future study should consider actual access and awareness; which supports are available per environmental and policy audits compared to employee awareness and use.

In worksites where multiple supports for PA were present, it is difficult to assess what cumulative effect these supports may have on employee PA and thus, to discern the power of specific supports in the context of a worksite that has an overall culture of health, wellness, and support for PA. To this end, our research team has published on the additive effects of specific worksite support pairs [38]. This work also considered the occupation and industry of participants, two variables not controlled for in the present analysis due to number of controls already incorporated. In another analysis, we found that the association of specific supports, e.g., stair prompts, were significantly associated with sedentary time within the educational/professional, service, and office/administrative support industries, but not associated with sedentary time in blue-collar industries, business, and the health care setting [39].

## Conclusion

This study provides insights into the utility of independent worksite supports for PA. Specifically, access to 15 of 18 worksite supports were significantly associated with PA and use of 11 of 14 supports were associated with PA. Findings from this study may assist employers interested in implementing worksite supports for PA in selecting the most appropriate and effective supports for their employees.

## Abbreviations

CDC: United States Center for Disease Control and Prevention
PA: Physical activity
ROI: Return on investment
